# Infectivity of deceased COVID-19 patients

**DOI:** 10.1007/s00414-021-02546-7

**Published:** 2021-03-05

**Authors:** Stefanie Plenzig, D. Bojkova, H. Held, A. Berger, F. Holz, J. Cinatl, E. Gradhand, M. Kettner, A. Pfeiffer, M. A. Verhoff, S. Ciesek

**Affiliations:** 1 Institute of Legal Medicine, University Hospital Frankfurt, Goethe University, Kennedyallee 104, 60596 Frankfurt am Main, Germany; 2 Institute of Medical Virology, University Hospital Frankfurt, Goethe University, Paul-Ehrlich-Straße 40, 60596 Frankfurt am Main, Germany; 3 Senckenberg Institute of Pathology, University Hospital Frankfurt, Goethe University, Theodor-Stern-Kai 7, 60590 Frankfurt am Main, Germany

**Keywords:** SARS-CoV-2, Autopsy, Post mortem examination, Pathology, Forensic medicine

## Abstract

The duration of infectivity of SARS-CoV-2 (severe acute respiratory syndrome coronavirus 2) in living patients has been demarcated. In contrast, a possible SARS-CoV-2 infectivity of corpses and subsequently its duration under post mortem circumstances remain to be elucidated. The aim of this study was to investigate the infectivity and its duration of deceased COVID-19 (coronavirus disease) patients. Four SARS-CoV-2 infected deceased patients were subjected to medicolegal autopsy. Post mortem intervals (PMI) of 1, 4, 9 and 17 days, respectively, were documented. During autopsy, swabs and organ samples were taken and examined by RT-qPCR (real-time reverse transcription-polymerase chain reaction) for the detection of SARS-CoV-2 ribonucleic acid (RNA). Determination of infectivity was performed by means of virus isolation in cell culture. In two cases, virus isolation was successful for swabs and tissue samples of the respiratory tract (PMI 4 and 17 days). The two infectious cases showed a shorter duration of COVID-19 until death than the two non-infectious cases (2 and 11 days, respectively, compared to > 19 days), which correlates with studies of living patients, in which infectivity could be narrowed to about 6 days before to 12 days after symptom onset. Most notably, infectivity was still present in one of the COVID-19 corpses after a post-mortem interval of 17 days and despite already visible signs of decomposition. To prevent SARS-CoV-2 infections in all professional groups involved in the handling and examination of COVID-19 corpses, adequate personal safety standards (reducing or avoiding aerosol formation and wearing FFP3 [filtering face piece class 3] masks) have to be enforced for routine procedures.

## Introduction

On March 11, 2020, the WHO (World Health Organization) declared COVID-19 (coronavirus disease) a pandemic [1]. The infection-causing severe acute respiratory syndrome coronavirus 2 (SARS-CoV-2) has been spreading around the world since its first description in December 2019 [2]. Since then, research into the new virus and associated disease has been carried out in many scientific disciplines. Post mortem examination including autopsies and subsequent analysis of samples plays an important role in understanding the pathogenesis of novel pathogens e.g. common thromboembolism in deceased patients infected with SARS-CoV-2 [3]. Based on these findings, therapeutic strategies could be adapted. In addition, SARS-CoV-2 RNA (SARS-CoV-2 ribonucleic acid) and viral particles could be detected in different organs [3, 4]. Subsequently, histopathological findings typical of COVID-19 were described [5].

During autopsy and even external examination of a corpse infected with SARS-CoV-2, a certain risk of infection for the medical staff carrying out the procedure must be assumed. In connection with the current pandemic, a forensic practitioner in Thailand was reported to have been infected during work, although it was stated later on that there was no scientific proof of infection taking place during autopsy [6, 7]. It is currently unknown how long SARS-CoV-2 positive corpses are infectious. Therefore, the objective was to examine swabs and tissue samples of SARS-CoV-2 infected deceased patients for their infectivity after various periods of time after death.

## Material and methods

Four corpses of deceased patients with proven SARS-CoV-2 infections were examined by performing a full autopsy (medicolegal standard of the procedure) with subsequent toxicological, histological and virological examination of the acquired specimen.

### Autopsy, histology, toxicology

Corpses were stored at 6–8 °C within about 12–24 h after death. Intervals between death and autopsy were 1, 4, 9 and 17 days, respectively. A full autopsy was carried out for all corpses. Tissue samples of the brain, heart, lungs, liver, spleen, pancreas, kidneys, adrenal glands, thyroid gland and paratracheal lymph nodes, as well as small intestine and colon, were taken for histopathological examination. The samples were fixed in buffered 4.5% formaldehyde solution, processed according to standard procedures and stained with haematoxylin-eosin. For toxicological analysis, cardiac blood, femoral venous blood and urine were obtained. Samples were analysed using gas chromatography and high-performance liquid chromatography-mass spectrometry.

For the acquisition of virological examination specimen, care was taken to avoid contamination during the process. Instruments were disinfected appropriately (Incidin^TM^ Plus 0,5 %; Ecolab Healthcare; Monheim am Rhein, Germany) before and after removal of the specimen and specimen were taken as soon as possible during the course of the autopsy. UTM^TM^ swabs (Hain Lifescience; Nehren, Germany) were taken from perioral, both palms of the hands and inner parts of the elbow, reflecting the areas with highest probability of virus particle deposition during coughing, as well as from the oropharynx, trachea and both lungs. Swabs from the lungs were obtained after disinfection of the organ surface (Octenisept®; Schülke & Mayr GmbH; Norderstedt, Germany) and subsequent incision with disinfected instruments (Incidin^TM^ Plus 0,5 %; Ecolab Healthcare; Monheim am Rhein, Germany). In addition, small tissue samples of the heart, lungs, brain, liver, spleen, pancreas, left kidney, thyroid gland, small intestine and colon were obtained and stored in physiological sodium chloride solution.

### Cell culture

Tissue samples and swabs were stored at −80 °C and thawed directly before preparation. Approximately 0.5 cm^3^ tissue specimen was dissected from original sample material and homogenised with 300 μL PBS (phosphate buffered saline). Both tissue homogenate and swabs were mixed with MEM (minimum essential medium) containing 1% FCS (foetal calf serum; Sigma-Aldrich; St. Louis, Missouri, USA), 3% Amphotericin B and 0.2% Primocin (InvivoGen; San Diego, California, USA). Swabs-inoculums were immediately transferred to Caco-2 cells (human colon adenocarcinoma cell line) seeded in 12.5 cm^2^ culture tubes, whilst tissue-inoculum was transferred to Caco-2 cells after removing cell debris by centrifugation and filtration with 0.45 μm filter. Cytopathogenic effect (CPE) was assessed daily for up to 7 days. After 7 days or when cell lysis occurred, supernatants were inspected for the presence of viral RNA by real-time reverse transcription-polymerase chain reaction (RT-qPCR). Total RNA from cell-culture supernatants was isolated using AVL buffer (viral lysis buffer) and the QIAamp Viral RNA Kit (Qiagen; Hilden, Germany) according to the manufacturer’s instructions. SARS-CoV-2 RNA was analysed by RT-qPCR using the Luna Universal One-Step RT-qPCR Kit (New England Biolabs; Ipswich, Massachusetts, USA) and primers targeting RNA-dependent RNA polymerase (RdRp):

- RdRP_SARSr-F2 (GTGARATGGTCATGTGTGGCGG)
- RdRP_SARSr-R1 (CARATGTTAAASACACTATTAGCATA).

### Real-time reverse transcription PCR

If there were remnants of original tissue/swab, specimen RNA was extracted out of the remaining original sample prepared for virus isolation using the QIAsymphony instrument (DSP Virus/Pathogen Midi Kit; Qiagen; Hilden, Germany). SARS-CoV-2 RNA was detected by using the Allplex^TM^ 2019-nCoV (Seegene; Seoul, Korea) according to the manufacturer’s recommendations.

## Results

The mean age of the four deceased persons included in this study was 81 years (min: 65 years, max: 88 years, male=3, female=1, Table 1). The post mortem interval (PMI) ranged from 1 to 17 days. Causes of death included adult respiratory distress syndrome (ARDS) in the course of COVID-19 as well as combinations of exacerbated pre-existing medical conditions and COVID-19 (Table 1). Relevant aspects of the (clinical) patient history of the four cases, if known, as well as the main results of the autopsy, histopathological examinations and toxicological tests, are presented below.

**Table 1:** Summary of the examined four cases including post mortem intervals (PMI)

Case No.	PMI/d	Age/y	Sex	Cause of death	Positive SARS-CoV-2 testing by PCR
1	1	65	M	ARDS due to COVID-19	29 days ante mortem
2	4	88	M	Pre-existing conditions combined with COVID-19	11 days ante mortem
3	9	88	M	Pre-existing conditions combined with COVID-19	19 days ante mortem
4	17	82	F	Pre-existing conditions combined with COVID-19	2 days post mortem

### Case 1

The 65-years-old male patient was admitted to the hospital with severe respiratory failure and suspected SARS-CoV-2 infection. A positive test result was gained 2 days after admission. During the course of his hospitalization he was intubated and ventilated, finally receiving extracorporeal membrane oxygenation (ECMO) treatment. He died in the hospital one month after admission. There were no known pre-existing medical conditions. Upon autopsy, a highly solidified dense lung tissue (weight left: 845 g, right: 1018 g) with fibrinous overlays of the visceral pleura, cardiac hypertrophy, coronary heart disease (CHD) and moderate atherosclerosis were noted. Histologically, a high level of diffuse irreversible alveolar damage with fibrosis, fibrin deposits of the visceral pleura and coronary atherosclerosis was identified. Close to small vessels, interstitial lymphocytic infiltrates were noted. The alveolar walls showed fibrotic reorganisation. Nuclei of alveolar macrophages appeared to be enlarged with prominent nucleoli. Toxicological tests of autopsy samples showed morphine, amantadine, midazolam, propofol, amiodarone and metabolic products of the aforementioned in non-toxic concentrations. Acute respiratory distress syndrome (ARDS) in the fibroproliferative phase of the disease due to COVID-19 was diagnosed as the cause of death.

### Case 2

The 88-year-old male patient was living in a geriatric institution affected by COVID-19. SARS-CoV-2 testing was carried out 11 days prior to death and showed a positive result. He was admitted to a hospital, where he died in the isolation ward. Known pre-existing medical conditions included chronic obstructive pulmonary disease (COPD), giant cell arteritis, mitral regurgitation and chronic kidney failure. Upon autoptical examination pulmonary oedema (weight left: 500 g, right: 780 g), COPD, cardiac hypertrophy, CHD, mitral insufficiency and granular renal atrophy were noted. Histologically, pronounced pulmonary oedema, patch-like lymphocytic infiltrates in alveolar septa, occasional microthrombi, signs of severe COPD and moderate coronary atherosclerosis were seen. Toxicological analysis of autopsy samples showed the presence of melperon in a non-toxic concentration. A combination of exacerbated pre-existing medical conditions and COVID-19 was the presumed cause of death.

### Case 3

The 88-year-old male patient was transferred to a geriatric ward after hip surgery, where he was then tested positive for SARS-CoV-2. His condition deteriorated over the next days with death occurring in the isolation unit of a hospital 19 days after the initial diagnosis. Pre-existing medical conditions included atrial fibrillation with pacemaker implantation, CHD, arterial hypertension and chronic cardiac and renal insufficiency. Upon autopsy solidified and partially oedematous lung tissue (weight left: 850 g, right: 875 g), COPD, cardiac hypertrophy, CHD, mitral insufficiency, signs of chronic right heart failure and severe atherosclerosis were seen. Histologically, severe pulmonary oedema, beginning diffuse alveolar damage with occasional hyaline membranes (exudative phase of the disease), desquamated alveolar macrophages partially with dysplastic nuclei, interstitial lymphocytic pneumonia and coronary atherosclerosis were seen. Toxicological tests yielded morphine in a non-toxic concentration. A combination of pre-existing diseases and COVID-19 was diagnosed as the cause of death.

### Case 4

The 82-year-old female patient had been tended by relatives after showing flu-like symptoms. Two days prior to death she began to suffer from fever, dyspnoea and cough. She was found on the floor in the living room inanimate by her husband. The death certificate stated 12:55 pm as the time of death and that no resuscitation efforts had been started after the initial contact due to existent signs of irreversible death. The woman had last been seen alive after 9 pm on the previous day. Pre-existing medical conditions included chronic heart failure, arterial hypertension, defibrillator implantation and schizophrenia. Not having been tested prior to death, a tracheal swab was taken 2 days after death, respectively 15 days before autopsy, which showed SARS-CoV-2 infection (cycle threshold: 21.6). Upon external examination, there were beginning signs of putrefaction with marbling of vessels, bloating of the body and mould infestation in the perioral and neck regions. Upon autoptical examination congested brittle lung tissue (weight left 570 g, right 585 g), CHD, cardiac hypertrophy, signs of chronic right heart overload, mitral insufficiency, defibrillator implantation and moderate general atherosclerosis were noted. Histologically, intraalveolar lung oedema with few lymphoid infiltrates in lung tissue, coronary atherosclerosis, beginning signs of putrefaction with post mortem bacterial overgrowth, starting cell fragmentation and weak staining of the nuclei in all organs were seen. Histological assessment was severely limited due to putrefaction. Toxicological tests revealed tramadol, melperon, quetiapine and olanzapine in non-toxic concentrations. Severe pre-existing medical conditions combined with COVID-19 were assumed as the cause of death.

### Results of virological examinations

Tissue samples of the lungs and swabs of some parts of the respiratory tract of case 2 und 4 showed infectivity in cell culture. In case 1 and 3, no cytopathogenic effect was observed in any sample. RNA of SARS-CoV-2 was detected in original tissue samples of the lungs in all four cases and in tissue specimen of the brain (case 3), small intestine and kidney (case 2). All results of post mortem virological examinations (cell culture and RT-qPCR from original tissue samples and swabs) are listed in Table 2.

**Table 2:** Cell culture and RT-qPCR from original tissue samples and swabs; SARS-CoV-2 RNA was determined in the lungs of all four cases and further in the tissue sample of the brain in case 3 and in samples of the small intestine and kidney of case 2. Most samples of the respiratory tract in cases 2 and 4 showed CPE in cell culture as a sign of infectivity. *CPE* cytopathogenic effect, *C*_*t*_ cycle threshold. Not tested: if there was no original tissue or swab supernatant sample left, no RT-qPCR was performed

Samples		Case 1		Case 2		Case 3		Case 4	
		CPE	PCR (*C*_t_)	CPE	PCR (*C*_t_)	CPE	PCR (*C*_t_)	CPE	PCR (*C*_t_)
Swabs	Perioral	No	Not tested	No	Not tested	No	Not tested	Yes	Not tested
	Right elbow (inner part)	No	Not detected	No	Not detected	No	Not detected	No	Not detected
	Left elbow (inner part)	No	Not detected	No	Not detected	No	Not detected	No	Not detected
	Right palm	No	35.55	No	Not detected	No	Not detected	No	Not detected
	Left palm	No	31.87	No	Not detected	No	Not detected	No	Not detected
	Oropharynx	No	Not tested	Yes	Not tested	No	Not tested	No	Not tested
	Trachea	No	Not tested	Yes	Not tested	No	Not tested	Yes	Not tested
	Right lung	No	Not tested	Yes	Not tested	No	Not tested	Yes	Not tested
	Left lung	No	Not tested	Yes	Not tested	No	Not tested	Yes	Not tested
Tissue	Brain	No	Not detected	No	Not detected	No	36.94	No	Not detected
	Thyroid gland	No	Not detected	No	Not detected	No	Not detected	No	Not detected
	Right lung	No	30.72	Yes	24.81	No	34.46	Yes	25.08
	Left lung	No	30.51	Yes	14.96	No	34.40	Yes	20.88
	Heart	No	Not tested	No	Not tested	No	Not detected	No	Not tested
	Liver	No	Not detected	No	Not detected	No	Not detected	No	Not detected
	Spleen	No	Not detected	No	Not detected	No	Not detected	No	Not detected
	Pancreas	No	Not detected	No	Not detected	No	Not detected	No	Not detected
	Left kidney	No	Not tested	No	34.83	No	Not detected	No	Not tested
	Small intestine	No	Not detected	No	20.99	No	Not detected	No	Not detected
	Colon	No	Not detected	No	Not detected	No	Not detected	No	Not detected

## Discussion

Studying the duration of the infectivity of SARS-CoV-2 in living patients (detection of infectious viruses in oral or nasal cavities) in a nursing home resident cohort with known onset of symptoms showed an infectious period of 6 days before to 9 days after onset of symptoms [8]. Singanayagam et al. had shown infectivity of living patients until 12 days after onset of symptoms [9]. In case 4 of this study, typical COVID-19 symptoms had been present for 2 days prior to death. In case 2, the patient was tested positive for SARS-CoV-2 11 days prior to death. Therefore, these two patients died approximately in the time interval that would have been considered infectious in living patients. In contrast, patients in cases 1 and 3 showed longer periods between the onset of symptoms/positive SARS-CoV-2 testing and occurrence of death (19 days and 29 days, respectively), typically not associated with infectivity in living patients. One of the factors decisive for the infectivity status of a corpse in COVID-19 thus seems to be the duration of the infection prior to death.

Another possible factor for the infectivity of cases 2 and 4 as opposed to cases 1 and 3 may be the viral load, which has been shown to decrease over time during the disease phase [10]. A correlation between viral load and infectivity has also been detected in cell culture studies [11]. Samples with a *C*_t_ (cycle threshold) value of 13–17 resulted in a positive cell culture, whilst at a *C*_t_ value of >34, viral infection in the cell culture could no longer be seen. In 50 % of the cases, viral infection was detected in samples with a C_t_ value of approximately 29.5 [11]. The *C*_t_ value in organ samples from the lungs of both infectious cases in this study ranged from 14.96 to 25.08. In cases 1 and 3, a significantly lower viral load (with *C*_t_ values of >30) was observed in samples of the lungs and an infectivity in the cell culture could not be detected accordingly. The tissue sample of the small intestine of case 2 showed no infectivity in the cell culture despite a comparably higher viral load (*C*_t_ 20.99), which may be attributed to sample-immanent inhibitors.

In case 4, the duration of infectivity was impressive. Despite early transportation to the cooling chamber after death, the corpse displayed external and internal putrefaction due to the 17-day-period between death and autopsy. Accordingly, microscopic examination showed accumulation of bacteria and beginning cell fragmentation. Tissue affected by putrefaction will typically undergo pH shifts and accumulation of metabolic products [12], which interestingly did not perturb the infectivity of SARS-CoV-2 in case 4. Here, the rapid onset of cooling may have played a decisive role. In studies examining the stability of SARS-CoV-2 in living patient samples, stability was decreased in samples stored in warm temperature environments [13]. Thus, post mortem infectivity and its duration may be substantially dependent on storage temperatures of a corpse.

In living patients, a transfer of SARS-CoV-2 by aerosols is currently being discussed [14]. Since aerosols (as well as droplets) may be generated during autoptical procedures (and possibly also during external examination of a corpse e.g. when moving the deceased), sufficient protective measures have to be enforced to minimise the risk of infection for medical personnel and for the police and other professionally involved groups. Since, according to the results obtained, cooling of the body may lead to a post-mortem infectivity duration of at least 17 days, which persists even in the presence of putrefaction-related changes, recommendations to reduce the risk of infection will have to be adjusted accordingly [15].

This study has some limitations. On the one hand, the low number of cases has to be mentioned; on the other hand, onset of initial symptoms and/or time of infection were not known in all cases. In this regard, admission to the hospital and the positive SARS-CoV-2 test results were used. In addition, the study does not reach an end point—thus, a post mortem infectivity of COVID-19 corpses of at least 17 days should be assumed despite the possible onset of putrefaction.

## Conclusion

Corpses of deceased COVID-19 patients have to be considered potentially infective for more than 2 weeks post mortem under typical cooling conditions. The infectivity is mainly dependent on the time interval between initial disease symptoms and the occurrence of death as well as the viral load and may be present even after the onset of decay.

Medical personnel (as well as other professional groups in the field) are therefore exposed to a certain risk of infection with SARS-CoV-2 during post mortem handling and examination of COVID-19 corpses—thus, adequate protective measures have to be enforced to reduce the risk of infection with SARS-CoV-2.
